# Allergic diseases of the skin and drug allergies – 2027. Successful treatment with intravenous immunoglobulin and prednisolone pulse therapy of toxic epidemal necrolysis and Stevens-Johnson Syndrome

**DOI:** 10.1186/1939-4551-6-S1-P113

**Published:** 2013-04-23

**Authors:** Arzu Didem Yalcin, Atil Bisgin, Gokalp Soykam, Cem Sezer, Ayse Akman

**Affiliations:** 1 Internal Medicine, Allergy and Immunology, Education and Research Hospital, Turkey; 2 Cancer Institue, Sweden; 3 Department of Internal Medicine, Allergy and Clinical Immunology-Intensive Care Unit, Antalya Education and Research Hospital, Antalya, Turkey; 4 Department of Pathology, Antalya Education and Research Hospital, Antalya, Turkey; 5 Dermatology Unit. Akdeniz University, Antalya, Turkey

## Background

SJS-TENs pathogenesis is not completely explained and its immunological symptoms are similar to graft versus host disease so; it is possible to say that SJS-TEN is a disorder of the cell-mediated immunity. We report a first case of patient with intracranial tumors who developed a cutaneous adverse drug reaction during lansoprazole and prophylactic anticonvulsants treatment.

## Methods

Our patient is a 64 year-old female, who had glioma and had been on post-op anticonvulsants therapy. On the 3rd day after she had an operation, lansoprazole was added to the therapy. After the first lansoprazole dose erythematous dusky red macules were occured in extremities and trunk and on the following day confluent purpuric lesions tended to run together in 95% of the whole body including scalp and, oral and genital mucosa. Nikolsky's Sign was positive on the skin. Body temparature was 38.4°C with heart rate of 146beats/min. GlascowComaScale was E1M1e, pupillary light reflex was 2/2+/+. SCORTEN was calculated as 5 and her biopsy resulted as TEN.

## Results

As a treatment, firstly fluid and electrolyte homeostasis and skin lesions were maintained. For daily nutritional requirements total parenteral nutrition was supplied. Human albumin and IVIG in dose of 400mg/kg were usedand pulse steroid therapy. She was discharged from the hospital on the 23rd day and followed in the clinical immunology unit after 2 months.

## Conclusions

On six day intensive care unit serum STRAIL level was 302 pg/mL and in blister fluid soluble TRAIL level was 603 pg/mL. Two months after dischange serum soluble TRAIL level was 546 pg/mL. We found that the amounts of soluble TRAIL were higher in TEN blister fluids than in serum at the same time and after two months. TRAIL and TWEAK were secreted by CD1a+ and CD14+ cells present in the blister fluids we studied.This result suggest that TRAIL could also be a mediators of keratinocyte cell death in SJS-TEN.

